# Enhanced dynamicity: evolutionary insights into amphibian mitogenomes architecture

**DOI:** 10.1186/s12864-025-11480-6

**Published:** 2025-03-17

**Authors:** Yi Xiao, Gengyun Niu, Haihe Shi, Zhenyu Wang, Renzeng Du, Yankuo Li, Meicai Wei

**Affiliations:** 1 Laboratory of Insect Systematics and Evolutionary Biology, College of Life Sciences, Jiangxi Normal University, Nanchang, 330022 China; 2 School of Computer and Information Engineering, Jiangxi Normal University, Nanchang, 330022 China; 3 Nanchang Key Laboratory of Microbial Resources Exploitation & Utilization from Poyang Lake Wetland, College of Life Sciences, Jiangxi Normal University, Nanchang, 330022 China; 4 School of Digital Industry, Jiangxi Normal University, Nanchang, 330022 China

**Keywords:** Mitogenome, Comparative genomics, Macroevolution, Gene rearrangement, Complexity, Evolutionary transitions

## Abstract

Mitogenomes are known for their structural dynamics and the complexity of their rearrangement patterns. However, their utility in metazoan comparative biology has not been fully exploited. Vertebrate mitogenomes are now sufficiently representative to allow the development of more advanced methods for comparing genome architecture. Furthermore, the relatively robust phylogeny of vertebrates at higher taxonomic ranks allows us to infer the patterns of genome evolution accordingly. In this study, using amphibians as an example, we performed data cleaning and manual annotation on 1777 samples from the NCBI and identified 88 rearrangement types, most of which were clade-specific. In addition, we quantified genomic changes in an evolutionary framework and obtained stepwise growth curves of the architectural changes. This study provides new perspectives for understanding the evolution of the mitogenomes in amphibians and is expected to facilitate the qualitative and quantitative development of mitogenomes research.

**Supplementary information:**

The online version contains supplementary material available at 10.1186/s12864-025-11480-6.

## Introduction

The architecture of mitogenomes, as the structural organization of genetic material, exhibits dynamic properties [1]. It displays remarkable plasticity in individuals [2, 3, 4], while taking radically various pathways in diverse lineages. The architectural diversity of the mitogenomes provides a noteworthy dimension for observing the complexity of mitochondria as an open system. This diversity encompasses a broad range of genomic rearrangements, including insertions, deletions, inversions, and transpositions, both within genes and in intergenic regions. These structural changes not only reshape the genome but may also influence its functionality, ultimately impacting species adaptability and evolutionary trajectories. For instance, significant innovations in metazoans, such as the emergence of multicellularity and bilateral symmetry, are associated with specific changes in mtDNA organization [5, 6, 7]. Despite the extensive knowledge on the dynamic and plastic nature of mitogenomes, a comprehensive understanding of the specific patterns and implications of genomic rearrangements within particular taxonomic groups remains limited. Therefore, a detailed investigation into the diversity of gene order within a focal taxon is crucial to elucidate how these structural variations contribute to adaptations and evolutionary pathways, filling a critical gap in our current understanding of mitogenome evolution.

Amphibians represent an intriguing model for such an investigation. Since 1997 [8], the extensive reorganization of their mitogenomes has attracted considerable interest. Features such as exceptional enlargement (up to 28.8k bp) [9], loss and gain of genes [10, 11, 12], and the stepwise nature of rearrangement [13], hint at the hidden diversity within amphibian mitogenomes. Furthermore, the long noncoding region or control region (CR) has merged as a “hot spot” for variation, with duplications, triplications and recombination events adding to the complexity [9, 10, 13, 14]. Across amphibian lineages, independent rearrangements have been documented, serving as phylogenetic signals of amphibian evolution [15]. However, further shuffling within the cluster [11], gene replications [16], and the presence of similar events in taxonomically distant groups complicate the picture [9]. Intraspecific rearrangement diversity further emphasizes the need for a comprehensive analysis [17, 18]. These known and unknown complexities underscore the urgency to expedite our understanding of evolutionary landscape of mitogenomes variations in amphibians.

In light of the decreasing costs of high-throughput sequencing technologies [19], and the advancements in long-read sequencing [9, 20, 21, 22], coupled with optimized assembly and annotation techniques [23, 24, 25], we are now poised to explore mitogenomes architecture in greater depth [26]. In this study, we specifically aimed to investigate the genomic architectural diversity of amphibians. To achieve this, we used NCBI2GO (unpublished) to clean the available data from 1777 samples representing at least 710 species, resulting in a high-quality dataset. Subsequently, employing qMGR [27] and qGO [28], we conducted qualitative and quantitative comparative studies within a phylogenetic framework, and found a phased growth trend of structural changes in the evolution of mitogenomes. By utilizing amphibians as a well-studied group with ample variables, we have demonstrated a scalable methodological framework that can serve as a conceptual blueprint, inspiring similar explorations across a wider array of taxonomic groups and furthering the reach of comparative genomics research.

## Materials and methods

### Cleaning and error checking

**Fig. 1:**
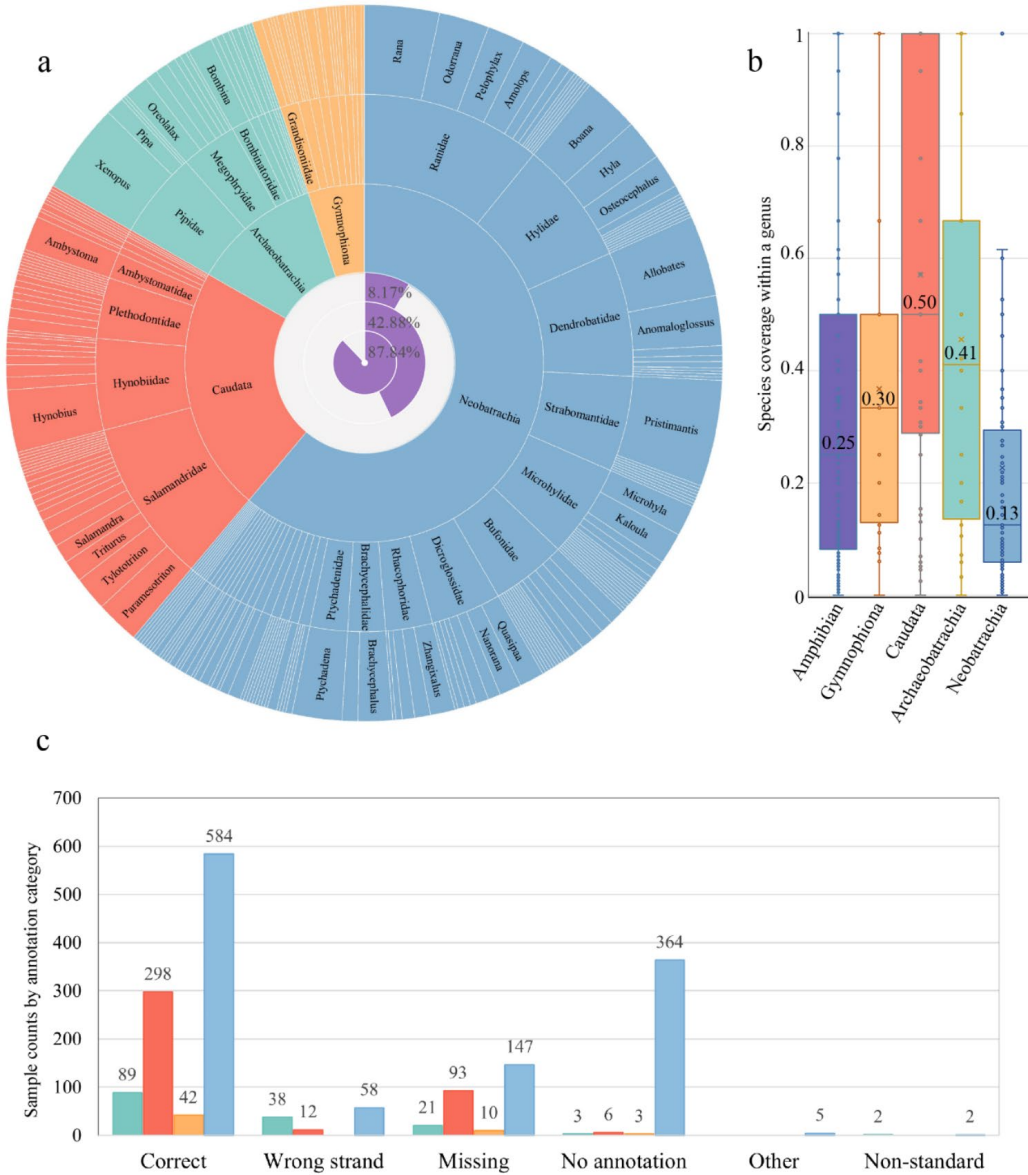
Amphibian mitogenome diversity and annotation status. **a.** Taxonomic distribution of amphibian mitogenomes. The outer circle of rising sun plot is based on the number of species in each genus, and the inner circle shows the coverage statistics for each rank under the AmphibiaWeb classification framework, which visualized from Table S3. **b.** The boxplot illustrating the species coverage of each genus within each taxon, showing the percentage of species coverage, which visualized from Table S3. **c.** The bar chart shows the annotation status of the 1777 amphibian samples, with the specific numbers labeled above each bar, which visualized from Table S4

Accurate annotation is often a challenge [29]. Current data suffer from several issues: a lack of annotations, a high error rate, and even errors within the curated RefSeq dataset [30, 31, 32, 33, 34, 35, 36]. Previously cleaned data cannot detect annotation errors at the source or are unusable due to inconsistent standardization across studies [7, 37, 38]. Therefore, this study employed a new tool, NCBI2GO (unpublished), which allows for re-annotation, followed by manual verification. To be specific, 2143 mitogenomes, including 366 from RefSeq [39], were retrieved from NCBI Organelle Genome Resources using the search string “Amphibia[ORGN] AND (mitochondrion[TITL] OR mitochondrial[TITL]) AND 10000:50000[SLEN] NOT (RNA [TITL] OR gene[TITL] OR product[TITL] OR mRNA[TITL] OR rRNA[TITL] OR misc_RNA[TITL] OR nuclear[TITL])” These mitogenomes were cleaned using NCBI2GO, and the gene order was extracted in a standardized format as described in the Abbreviations section. Out of 1777 samples, 376 with missing or invalid annotations (see Fig. 1c and Table S4), and those with unconventional gene orders, were processed through the re-annotation module relying on MITOS [32]. The intergenic length was calculated based on the location information to identify the control region (CR) using a threshold. It should be emphasized that the annotation of the two *trnL* and two *trnS* followed the designation by Boore [5], which is also the processing method utilized by MITOS.

### Taxonomic reconciliation

Coherence between species names and their parent taxa is central to comparative analysis at higher taxonomic levels. The NCBI taxonomy was checked against an authoritative list of accepted species. We chose to initially base this comparison on AmphibiaWeb (AW), which contains 77 families with 569 genera and 8689 species downloaded on January 13, 2024 [40].

The 1777 samples were first de-duplicated based on NCBI taxonomy, yielding a list of 97 unidentified species samples (17 with “*cf.*”, 79 with “*sp.*”, and 1 unisexal lineage), 1 hybrid, and 40 with “*aff.*”, 695 species, and 13 subspecies. Matching of the last three of the above five cases returned the scientific names of 716 species, and the remaining 32 were manually matched to the GBIF Backbone Taxonomy [41]. After the final deduplication, our taxonomic matchup placed 1777 samples into 710 species of 244 genera and 65 families and form a comprehensive dataset as showed in Table S1, S2, S3, S4.

### Quantification of mitogenome rearrangements

The mitogenomic structural changes can be analyzed quantitatively by calculating the pairwise distance between the rearrangements and the ancestral organization. We applied both the qMGR and qGO algorithms to the above dataset. While both aim to quantify the rearrangement frequency (RF) of each individual gene and the rearrangement score (RS) of each mitogenome, qMGR is alignment free, which calculates the RF of a given gene by accumulating the changes in the two nearest neighboring genes. However, qGO relies on homology alignment to directly calculate the RF of the target gene. It also supports weighting, meaning that breakpoints need to be predefined. Following the widely accepted processes of replication [42] and transcription [43, 44], we set two breakpoints to divide the circular genome into two intervals. Subsequently, genes within each interval were manually aligned across all types as in Table S5 and Supplementary File 10. For the RF (Table S6), types 1 and 21 were selected as references, while for RS, a matrix for all types was generated without the need for a reference (Table S7 and Fig. 3a), and RS of each types relative type 1 was collected to visualize the RS distributions across taxon (Table S8). To investigate the drivers of diversification, we analyzed the correlation between species richness and total RS across families (Table S1). Using LOESS smoothing for non-linear regression, we generated visualizations to identify families deviating from the general pattern (Fig. 3b).

### Evolution of mitogenomic structures across amphibians

**Fig. 2:**
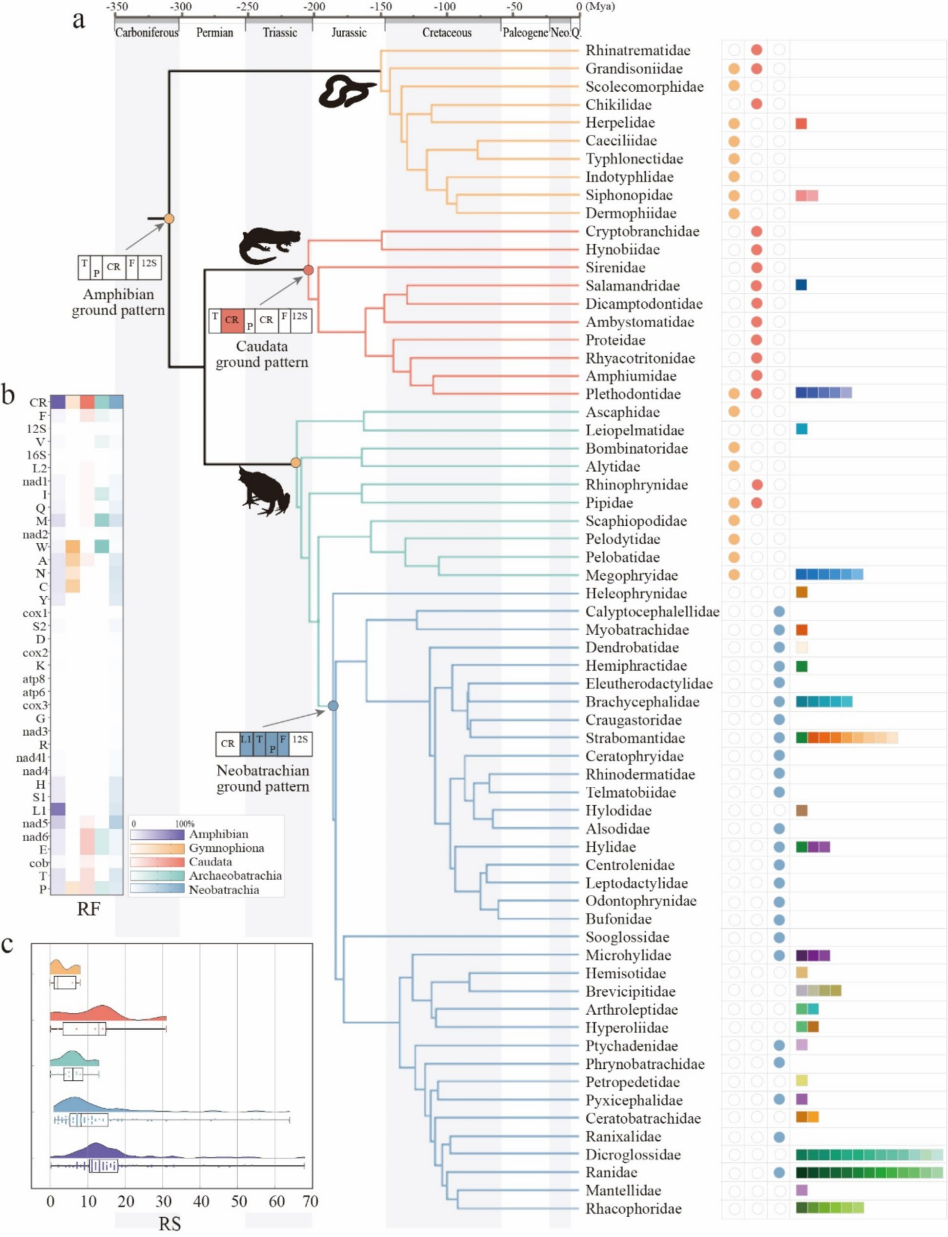
Evolutionary dynamics of mitogenomic architecture in Amphibia. **a**. The diagram illustrates 88 different types of structural variation in the amphibian mitogenome characterized by different color-coded blocks. Three ground patterns are represented by circular elements in orange, red and blue, corresponding to specific branches. In addition, the derived variations are represented by square elements with unique colors. **b**. Heatmap based on RF (Rearrangement frequency) reflecting the intensity of structural variation in mitochondrial genomes within different taxonomic units. **c**. Cloud-rain diagram and box plot comparing the RS (Rearrangement score) of 88 gene rearrangement types distributions across taxonomic units

**Fig. 3:**
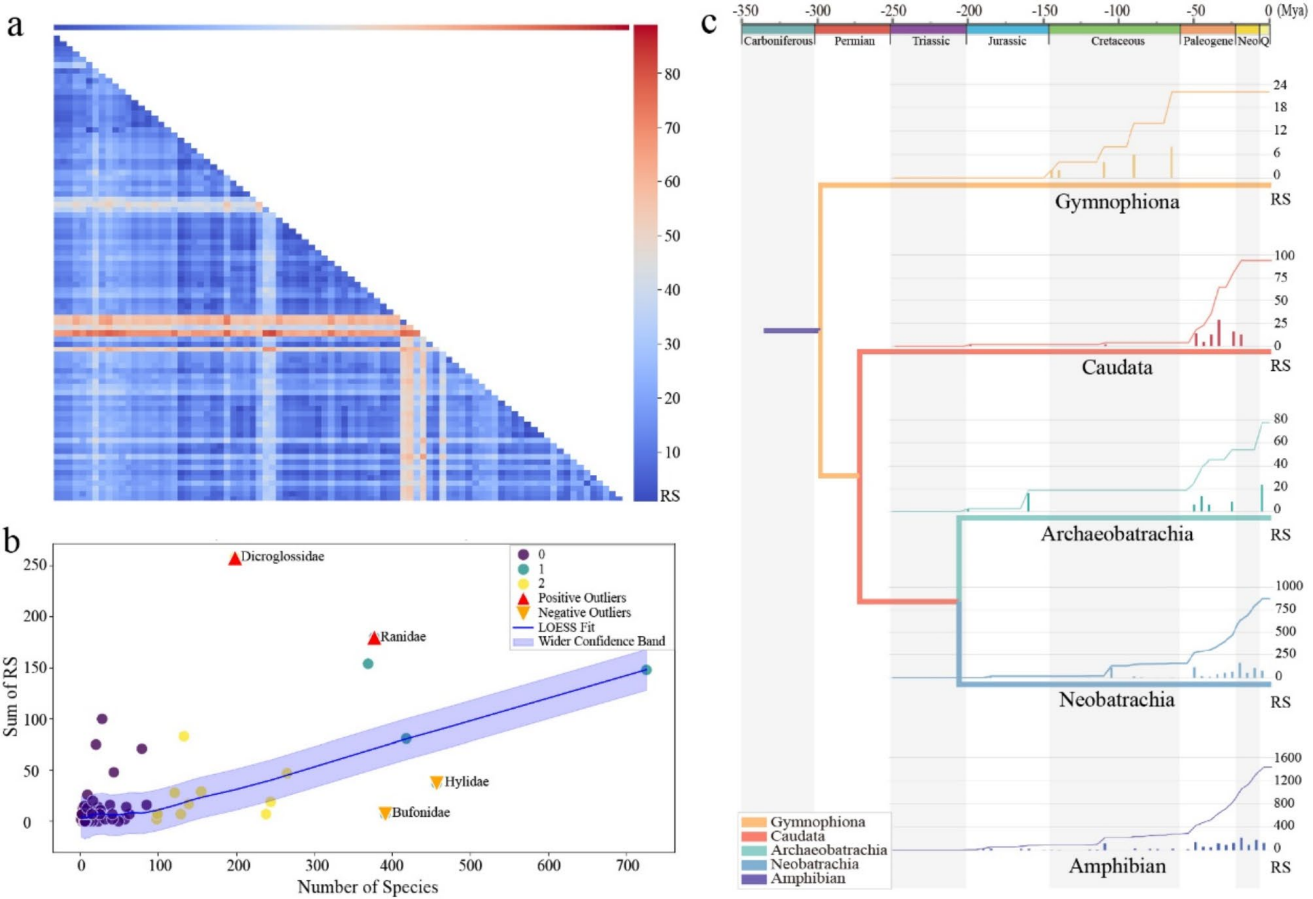
Relationship between mitogenomic rearrangements complexity, species diversity, and evolutionary dynamics in Amphibia. **a**. Heatmap showing the matrix of rearrangement scores (RS) across 88 gene rearrangement types with darker colors indicating greater rearrangement changes, which visualized from Table S7. **b**. Correlation between rearrangement complexity and species diversity, with LOESS smoothing applied to highlight trends and identify outliers, which visualized from Table S1. **c**. The changes in rearrangement scores (RS) over time for taxonomic units, reflecting the dynamics of changes in their mitochondrial genome structure, which visualized from Table S8

We employed the time-calibrated molecular phylogeny of Jetz and Pyron [45] as the foundational framework. This phylogeny was updated by incorporating 12 recently revised families (Hynobiidae, Ambystomatidae, Proteidae, Rhyacotritonidae, Amphiumidae, Plethodontidae, Ascaphidae, Alytidae, Rhinophrynidae, Scaphiopodidae, Megophryidae, Limnodynastidae), while 7 taxa (Odontobatrachidae, Conrauidae, Nyctibatrachidae, Ceuthomantidae, Cycloramphidae, Batrachylidae, Allophrynidae) were pruned due to insufficient mitogenomic data coverage. All types are first projected to the phylogeny, and the ancestral type for the diversity is inferred to be the relative outgroup (Fig. 2c). Subsequently, the phylogeny was sliced into 34 time intervals, each spanning 5 million years. For each interval, the gene order types were inferred for all nodes within the interval, and their RS values were accumulated to obtain the total RS for that interval. Next, we presented the total RS values for each time interval by taxon in a bar chart, and illustrated the cumulative change trend of these total RS values over time using a line graph (Fig. 3c).

## Results

### Overview of mitogenomic organization in amphibians

A significant number of persistent and stubborn errors in the annotation were identified and reviewed [36]. Error types include but are not limited to abnormal reading direction (strand), erroneous gene designations, missing gene or other feature annotations, mistaken identity of *trnL1*/*trnL2* and *trnS1*/*trnS2*, and inconsistencies in gene names. The most misleading situation is when the errors are included in RefSeq, such as *trnY* being on the opposite strand in NC_028278 and one of the *trnM* gene being missing in NC_030627. After data cleaning, error checking and taxonomic reconciliation, we obtained a dataset containing 1777 samples representing 710 species in 244 genera under 65 families with family-level coverage of 87.84%, genus-level coverage of 42.88%, and species-level coverage of 8.17% relative to the AmphibiaWeb classification system (Fig. 1a), yielding 88 gene rearrangement types (Table S1). Species coverage within each genus was variable, with a mean of 35% and a median of 25%. Among them, Caudata was the richest (mean 58%, median 50%), and Neobatrachia was the least abundant (mean 23%, median 13%) (Fig. 1b and Table S3).

The unexpected complexity and variability among amphibian mitogenomes were revealed (e.g., Fig. 2c and Table S2). The major lineages of both Gymnophiona and Archaeobatrachia, as well as a few Caudata, were identical to the typical vertebrate gene order (orange circle in Fig. 2a), which was named the Amphibian ground pattern herein and labeled type 1 in Table S2. In addition, both Caudata and Neobatrachia have their own autapomorphies. The Caudata ground pattern (type 2, red circle) features one more CR derived between *trnT* and *trnP*. The Neobatrachian ground pattern (type 21, blue circle) has a strongly rearranged gene order, involving the long-term translocation of *trnL1* to form the *LTPF* tRNA gene cluster located upstream of the 12S rRNA gene [46]. In contrast to all 10 Caudata families, which have consistently retained the ground pattern among their members, only 24 of 35 Neobatrachia families show the Neobatrachia ground pattern. Among these, 9 families have evolved new types. The remaining 11 families exhibited completely derived types, leading to a total of 69 rearranged types. This makes Neobatrachia the most variable group, followed by Archaeobatrachia with 10 types, Caudata with 8, and Gymnophiona with 5, as shown in Fig. 2a.

According to the RF scores from qGO (Table S6), the most common rearrangement events observed in this study involved CR (169), *trnL1* (142), and *nad5* (52). Inversions are rare relative to translocations. Notably, consistent with findings from long-read sequencing, multiple rearrangement types involved duplication of genes or gene clusters, as well as repetition of CR or a combination of both [47]. Among them, *trnM* duplications were the most common, followed by *trnP* duplications, as detailed in Fig. 2b and Table S2.

### Mitogenomic structural changes across four lineages

Mosaic evolution and lineage specificity coexisted throughout mitogenome evolution in amphibians. In particular, the ground patterns of vertebrate (*n* = 162) and Caudata (*n* = 415) are shared among distantly related species of Gymnophiona, Caudata, and Archaeobatrachia. However, both patterns are notably absent in Neobatrachia. Moreover, the 67 derived types that evolved from the Neobatrachia ground pattern (*n* = 756) are unique to this lineage and are not observed in the other three lineages. These observations challenge our understanding of the organization of the ancestral amphibian mitogenome. Both Rhinatrematidae and Ichthyophiidae (*Uraeotyphlus* only), the two most basal families of Gymnophiona, possess the Caudata ground pattern [48]. Given the rearrangements found in Caudata and Archaeobatrachia, it is difficult to rule out the possibility that the ancestral state of the amphibians was a Caudata ground pattern. However, the majority of Gymnophiona, with the exception of the aforementioned families, exhibit this vertebrate pattern. Therefore, applying the principle of parsimony, the most plausible hypothesis is that the ground pattern of amphibian mitogenomes is a vertebrate pattern.

Almost all mitogenomic structural changes in Gymnophiona are genus specific, except for *Boulengerula*, which has interspecific variation, with one of the two species having a duplicated *trnP* (Type 3). There are also two types (type 4 and type 5) concentrated in the *WANCY* tRNA gene cluster that are restricted to two genera in Siphonopidae. The Caudata ground pattern is shared by all 10 Caudata families, yet two of them have derived taxa. Among the 168 Caudata species examined, there is intrageneric differentiation in the genus *Tylototriton*, and two of these species are type 11, involving the expansion of the CR and its flanking genes [14]. The seven Plethodontidae species from *Tylototriton*, *Aneides*, *Hydromantes* and *Plethodon* are all intragenerially diverse to types 6–9, and the single-sample *Stereochilus* is type 10. Archaeobatrachia comprises four successively evolved lineages, all of which can be traced back to the ancestral amphibian type. Two out of four genus of *Pipidae*, *Pipa* and *Xenopus*, possessed both Type 1 and Type 2. Derived types 20 (swaps of *nad6*-*trnE* and *cob*-*trnT*) were found in both examined species of Leiopelmatidae. The more diverse Megophryidae shows additional seven derived types occurring in *Leptobrachium* (Type 14), *Leptobrachella* (Type 13) [49, 50], *Scutiger* (Type 17) [51] and *Oreolalax* [49, 52, 53], which intragenetically diversified into five derived types. These changes primarily involve the duplication of *trnM* [46] and the remote transposition of *trnW*.

There is very high variation in the genomic structure of Neobatrachia. Whether derived in terms of majority rules or the status of the outgroups, the ancestral type should contain a unique rearrangement in which *LTP* tRNA gene cluster located between the CR and *trnF*. In addition to type 21, which is shared by 24 families, considered to be the ground pattern, there is another type shared by four distant families (type 48), which differs from the ground pattern only by an additional CR between *nad5* and *nad6*. Another pair of sister groups shares type 27, which has an additional interchange of *trnA* and *trnN* on top of the ground pattern. The remaining 66 types other than these three are unique to a given family, meaning that they are no longer shared between families [46]. The clade comprising Dicroglossidae [17, 46, 54, 55, 56], Ranidae [55, 57, 58, 59, 60, 61, 62, 63] and Rhacophoridae [64, 65, 66] exhibits the strongest changes, encompassing nearly half of the derived types (32 in total) with *Breviceps*, *Hyperolius*, *Ptychadena*, *Cornufer*, *Limnonectes*, *Amolops*, *Nidirana*, *Odorrana*, *Rana*, *Polypedates*, *Nanorana*, *Quasipaa*, and *Nanorana* presenting intrageneric changes and the latter two even presenting intraspecific changes [17, 18]. Nested within this clade, Mantellidae and Ranixalidae each harbor family-specific types. Other intrageneric changes occur in *Cornufer*, *Ptychadena* [67], *Hyperolius* [9], *Breviceps* [9, 68], *Ischnocnema* [69], *Brachycephalus*, *Boana*, and *Bokermannohyla* and reach an extreme of nine types in *Pristimantis*. The changes are varied and involve almost all regions of the mitochondria. The most notable variable regions involved the *trnA* and *WANCY* tRNA gene cluster [70], in particular, multiple OLs were identified in the cluster [71].

### Quantitative analysis of mitogenomes changes across amphibians

The hotspot is depicted in Fig. 2b on the heatmap plotted against the relative frequency (RF), detailed in Table S6. Each amphibian group is represented by a different color, with purple indicating the entire amphibian group. The gradient of color shading reflects the relative RF score. Among all the genes, *trnL1* exhibited the highest score due to its long-range translocation across various amphibian taxa. However, when examined at a finer taxonomic level, *trnL1* rarely undergoes changes within that specific taxon. It should be noted that hotspots differ between taxa. For instance, Gymnophiona primarily experiences changes in the *WANCY* tRNA gene cluster, while Archaeobatrachia scores highest for the flanking genes of *nad2*. Neobatrachia displayed significantly more variable regions than did the other taxa, with almost all the genes exhibiting some degree of rearrangement except for *cox1*,* cox2*, and *trnG*, which had RF scores of 0. Notable hotspots common to all three taxa may be confined to the region between *nad6* and *trnF*, including CR [16].

Further analysis of the RS distribution (Fig. 2c and Table S2) highlighted the significant variation in mitogenomes architecture among amphibians. Gymnophiona as the most primitive group, exhibited the lowest median RS intensity, suggesting a relatively low level of genomic change. Caudata has a greater median RS than Archaeobatrachian, possibly due to *Pyxicephalus adspersus*, which undergoes strong rearrangement with gene duplication, resulting in 49 genes [12]. This extreme value elevates the overall distribution profile with score peaks at 68 (qGO) and 46 (qMGR). However, disregarding this extreme value, both taxa exhibited relatively uniform distributions of RS that were generally greater than those observed in Gymnophiona. It is worth noting that although both Caudata and Archaeobatrachia had a minimum RS value of 0, considering that type 1 in Cadauta evolved from ancestral type 2, this score varied when type 2 was changed to the reference. The multimodal distribution observed in both Archaeobatrachia and Neobatrachia indicates heterogeneity, potentially suggesting divergent evolutionary directions. A more intriguing alternative inference concerns the emergence of Neobatrachia, which disrupts continuity and gives rise to fluctuations in Archaeobatrachia. Neobatrachia, as the most recently evolved lineage, stands out for its high RS values and several extreme values. There are eleven values above 20, a magnitude that far exceeds that of any other taxon, suggesting that it may have evolved in an aggressive way, avoiding the fatal decrease in evolutionary potential. This is consistent with the evolutionary pattern observed in other vertebrates, where more recently evolved taxa tend to have greater complexity. The extremely high RS may also indicate that the taxa have undergone rapid adaptive radiation or evolutionary innovation, resulting in significant increases in complexity.

Building on the quantitative analysis of mitogenomic changes across amphibians, we further explored the relationship between species richness and rearrangement complexity (RS) to uncover potential drivers of diversification (Table S1 and Fig. 3b). Our analysis revealed that the majority of data points are concentrated in the lower-left corner of the visualization, indicating that families with low RS scores generally exhibit lower species richness. As RS scores increase, species richness shows an overall upward trend, albeit with notable fluctuations and deviations. Specifically, we identified Dicroglossidae and Ranidae as positive outliers, characterized by exceptionally high RS scores but unexpectedly low species richness. This suggests that these families possess rearrangement diversity beyond typical levels, potentially reflecting unique evolutionary or ecological adaptations. Conversely, Bufonidae and Hylidae emerged as negative outliers, displaying high species richness despite very low or absent rearrangement events. This discrepancy may indicate alternative mechanisms driving diversification in these families, such as ecological niche partitioning or adaptive plasticity.

### Evolutionary dynamics underlying patterns of diversification

By aligning the RS of each taxon with the time of origin of its corresponding branch and aggregating the RS values over time, a growth curve can be generated (Fig. 3c and Table S8). A comparison between the growth curves for all amphibians and those for each individual taxon revealed a consistent pattern of stepwise growth over time. This suggests that there were distinct periods of rapid expansion followed by relatively stable phases in amphibian evolution.

Interestingly, while the absolute values of RS exhibited considerable variation across taxa, they demonstrated remarkably consistent patterns of increase, except for Gymnophiona, which consistently experienced a step change during the Cretaceous period. This indicates that Gymnophiona underwent unique evolutionary events or adaptations during this specific period. Furthermore, when examining the remaining three branches, it becomes evident that they all display a rapid increase with nearly comparable slopes soon after the beginning of the Paleogene Period. This sudden surge in their RS values is preceded by an extended period of stagnation and a minor, limited magnitude increment occurring around or prior to the mid-Cretaceous epoch. The long periods of silencing in the early clade may indicate an underestimation of potential diversity for various reasons, such as extinctions associated with specific rearrangements.

## Discussion

### Unraveling the complexity: mitogenomes variability in amphibians

The dogma that mitogenomes in vertebrates are frozen has long been broken, but vertebrates are still considered subphyla with probably the lowest variability in mtDNA gene content and gene order, with disparities thought to have a limited taxonomic distribution. This frosted glass exists mainly because of uneven sampling [72], a high percentage of erroneous annotations [33] and problematic mitogenomes [34], as well as the omission of duplicated regions [73]. During the last few years, the advent of high-throughput sequencing techniques has increased the number of sequenced mitogenomes. Vertebrates, in particular, account for more than half of all Metazoa [7]. Specifically, for amphibians, an adequate representation of 88% of the families is sufficient to infer consensus mitogenome characterizations of different taxa and to fully recognize exceptions that lie beyond this consensus. The present study revealed that 43% of the amphibian genera examined had at least one available mitogenome. However, the data still suffer from taxon bias. In the case of Gymnophiona, mitogenomic structural changes often occur at the genus level. The lack of novel rearrangements, despite a genus-level coverage of 82%, suggests data saturation. Conversely, although the number of Archaeobatrachia mitogenomes exceeds that of Gymnophiona mitogenomes, changes could occur within genera even within species, suggesting that as species diversity increases, the emergence of new types of mitogenomes is possible. In other words, the existing data for Archaeobatrachia may be insufficient [49].

The mitogenomes, traditionally considered highly conserved, exhibits unexpected dynamism, with 88 observed types in our study. This diversity, however, is likely an underestimate, suggesting that the complexity of the mitogenomes extends beyond current comprehension, especially when considering regions of high variability [13, 47] that were excluded from this dataset due to stringent search criteria. This highlights the critical importance of reference selection in quantitative analyses, as exemplified by the observation of *trnL1*: while its RF score is remarkably high (142) relative to type 1, it becomes negligible (only 25) when compared to type 21. In a phylogenetic framework, structural diversity is sensitive to the grain of phylogenetic resolution. Specifically, the differences between major types (type 1 and type 2) in amphibians, birds and reptiles [23, 74] hinge on the presence of an additional CR between *trnT-P*, and Neobatrachia shows phylogenetic constraints from ancestral type 21. In contrast to the path-dependent evolutionary trajectories described above, fine-grained taxonomic diversifications appear to be stochastic and unpredictable, underpinning the individualized nature of mitochondrial evolution within specific families [75], as depicted in Fig. 2a. These changes involve all kinds of components, including *trnA*, protein-coding genes, rRNAs, and the CR, occurring regardless of the gene position relative to the CR and affecting both strands.

Based on such observations, it is reasonable to hypothesize that a variety of mechanisms can drive mitogenome structural changes, extending beyond the duplication-random loss (DRL) model traditionally invoked to explain genomic alterations [77, 78, 79]. The DRL model, while useful in explaining certain types of genomic changes at the individual level, may not fully account for the broad variability observed across different lineages [46, 80] or proclivity, such as nested copies of duplicated segments [68]. This raises critical questions about the underlying mechanisms that confer flexibility and robustness to mitogenome evolution, thereby enhancing its evolvability and adaptability across diverse lineages [37, 76]. Based on this insight, it is imperative to re-evaluate whether, and under what conditions or to what extent, rearrangements may exhibit convergence.

### An asynchronous symphony of the episodic architecture variation

Complexity often emerges from an intricate dance of genetic and environmental factors, resulting in the quantification of features that exhibit fluctuations across multiple dimensions. Through the use of RS and RF as proxies for measuring complexity, genetic innovations can be traced step by step, and amphibian evolution dynamics can be linked to genomic structure evolution. As shown in Fig. 2c, primitive taxa tended to exhibit simpler structures and lower levels of RS, while derived taxa, on the other hand, showed a significant increase in RS across several levels. This increase in complexity suggests that the genomic structure is able to evolve, enabling the exploration of new strategies and facilitating diversification [81]. In other words, evolvability evolved [82]. The case of Archaeobatrachia sheds further light on how this trend impacts specific groups within these taxa. The fluctuating RS suggests that Archaeobatrachia may have undergone a phase of complexity reduction or macroevolutionary freezing [83], which is precisely a consequence of the evolution of evolvability [84].

Rearrangement frequency (RF) helps us understand complexity by showing that genes or gene clusters within a genome may undergo independent evolution [85, 86]. The *trnW* gene was used as an example. Although its overall RF appears normal across various amphibian groups, a zoomed-in examination revealed heterogeneity. Within Archaeobatrachia, all types involved *trnW* shifts, resulting in a remarkably high RF. In contrast, RF rapidly decreased in Gymnophiona but reached 0 in Caudata. The heterogeneous behavior of gene rearrangement challenges the notion that the mitogenome evolves as a cohesive, inseparable evolutionary unit. It becomes evident that the way a genome evolves depends on the distinct patterns of independent evolution present among the different genes or gene blocks that a particular genome contains [87]. It is noteworthy that the RF scores derived from the qMGR and qGO algorithms exhibit similar overall trends. However, in the qMGR analysis, the scores for *trnH*, *trnE*, *trnS2*, and *trnV* are underestimated. This discrepancy arises from the algorithm’s inherent limitation in appropriately assigning scores to internal units within gene clusters that undergo collective transposition, as detailed in [28]. Additionally, due to ambiguities in the original annotation of *trnL1* and *trnL2*, the scores for these genes in the qMGR analysis are also biased (Supplementary File 9).

By examining changes in RS over time, it is possible to trace the increasing complexity of genomic structures and to infer the reasons for this complexity. As shown in Fig. 3c, across multiple time intervals, we observe a pattern where periods of stasis are punctuated by brief bursts of rapid evolution. The concept that the rate and pattern of evolutionary change are closely linked to selective pressures at the lineage level is a longstanding and vital part of macroevolutionary theory [88], and this concept also applies to mitogenomic evolution [89]. However, the episodic increase in the number of salientians (anurans and caudates) showed a similar pattern at significant timescales. The first increase, which was very small and occurred at the beginning of the Jurassic, was followed by an increase in limited growth near the Jurassic and Cretaceous boundaries, and a third significant increase occurred just after the K-Pg line, overlapping with the rapid diversification of species-rich clades [90, 91]. Between these periods of growth are long periods of stagnation, which may reflect temporarily rising ‘challenging times’ for diversification of mitogenomes, possibly due to evolutionary constraints. The generalizability of this pattern across different amphibian clades highlights the dynamic nature of mitogenomes and the intermittent nature of the evolutionary events that shape their structure. Episodic mitogenomic evolution is also evident in other metazoans, such as fish and invertebrates [92] In these organisms, drastic changes in mitogenomic structure have been linked to episodes of adaptive radiation [93] or extreme environmental adaptation.

The complexity and evolvability of amphibian mitogenomes structure could be shaped over evolutionary time by various external forces [94]. These forces include not only the deterministic effects of evolutionary events but also the stochastic influence of random changes. This interplay of factors has endowed mitogenomes with a capacity for adaptation and innovation that exceeds the predictions of traditional evolutionary models. This adaptability is particularly evident in the heterogeneous nature of mitogenomic evolution across amphibian families, where significant deviations from general trends highlight the intricate processes driving diversification. The evolvability of mitogenomes through gene rearrangements highlights the dynamic role of genome structure in shaping evolutionary trajectories. These patterns are consistent with broader evolutionary dynamics in vertebrates, where more recently derived lineages often exhibit heightened genomic complexity and adaptive potential. Beyond serving as repositories of genetic information, mitogenomes actively participate in evolutionary processes by responding to selective pressures and environmental challenges through structural changes. Further investigation into the ecological and evolutionary contexts of outlier families should prioritize expanding taxonomic coverage to include underrepresented lineages, aiming to unravel the functional and ecological implications of mitogenomic rearrangements.

## Conclusions

We collected and curated all available mitogenomes for amphibians and discovered the unexpected dynamism of their architecture. The quantification of structural changes reveals the episodic pattern and highlights the diversity in terms of lineages and genes. This study sheds light on the mechanisms driving genome evolution and underscores the importance of genome structure in shaping the evolutionary fate of organisms. We challenge the simplistic view of genetic structural changes as discrete, qualitative units and emphasize more specific quantitative descriptions of patterns of genome structural evolution.

## Electronic supplementary material

Below is the link to the electronic supplementary material.

Supplementary Table S1

Supplementary Table S2

Supplementary Table S3

Supplementary Table S4

Supplementary Table S5

Supplementary Table S6

Supplementary Table S7

Supplementary Table S8

Supplementary File 9

Supplementary File 10

## Data Availability

The data sets presented in this study can be found in online repositories. The names of the repository/repositories and accession number(s) can be found at: https://figshare.com/account/home#/projects/196681. This study exclusively uses publicly available sequencing data from NCBI (accession numbers listed in Supplementary Table 4).

## Abbreviations

mitogenome: Mitochondrial genome
mtDNA: Mitochondrial DNA
tRNA, trn: Transfer ribonucleic acid
trnL1: trnL (CUN)
trnL2: trnL (UUR)
trnS1: trnS (AGY)
trnS2: trnS (UCN)
rRNA: Ribosomal ribonucleic acid
mRNA: Message ribonucleic acid
bp: Base pair
nad1-6, 4l: NADH dehydrogenase subunit 1–6, 4 L
cox1-3: Cytochrome oxidase subunit I-III
atp6, 8: ATPase subunit 6, 8
RS: Rearrangement score
RF: Rearrangement frequency
qMGR: Quantifying mitogenome rearrangements
CR: Control region
OriL: The origin of light strand replication
OriH: The origin of heavy strand replication
