# Supplementary material for: Enhanced dynamicity: evolutionary insights into amphibian mitogenomes architecture
Source: BMC Genomics. 2025 Mar 17;26:261. doi: 10.1186/s12864-025-11480-6 (PMC11917051; doi:10.1186/s12864-025-11480-6)
Supplement: Supplementary file 9 [file 12864_2025_11480_MOESM9_ESM.docx]

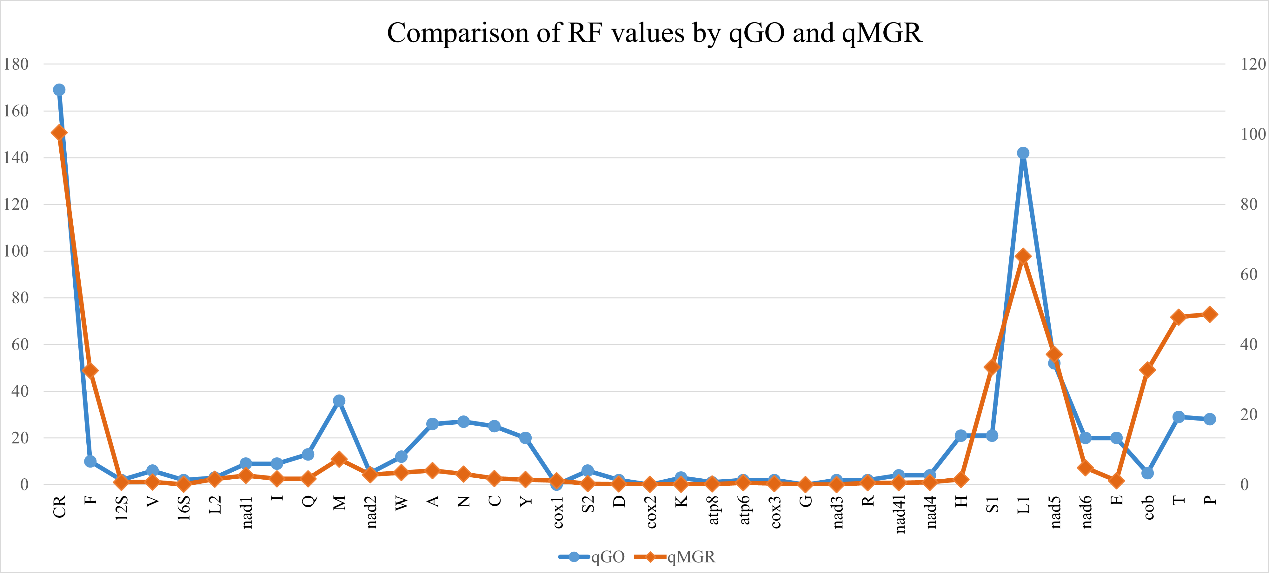


Comparison of rearrangement frequency (RF) values for different genes in amphibians, calculated using qGO (blue line) and qMGR (orange line) methods. The x-axis represents the genes, and the y-axis represents the RF values.
